# Supporting the drive for net zero by decarbonising general practice – A longitudinal study protocol

**DOI:** 10.3310/nihropenres.13833.1

**Published:** 2024-12-24

**Authors:** Ana Raquel Nunes, Helen Atherton, Frederik Dahlmann, Abi Eccles, Olivia Geddes, Michael Gregg, Sue Jowett, Florence Karaba, Laura Nelson, Rachel Spencer, Helen Twohig, Jeremy Dale

**Affiliations:** 1 University of Warwick Warwick Medical School, Coventry, England, UK; 2 University of Southampton School of Health Sciences, Southampton, England, UK; 3 Warwick Business School, Coventry, England, UK; 4 University of Oxford Medical Sciences Division, Oxford, England, UK; 5 PPI Representative, NA, UK; 6 University of Birmingham Health Economics Unit, Birmingham, England, UK; 7 NHS Coventry and Warwickshire STP, Rugby, England, UK; 8 Keele University School of Medicine, Keele, England, UK

**Keywords:** climate change; carbon footprint; primary care; general practice; longitudinal study; mixed methods.

## Abstract

**Introduction:**

The urgency to address climate change and reduce carbon emissions in healthcare, highlighted in the United Kingdom (UK) by the 'Delivering a 'Net Zero' National Health Service (NHS)' report, emphasises the need for healthcare organisations to adopt and integrate sustainability practices. General practice is recognised as a major source of greenhouse gas emissions, but evidence on achieving effective decarbonisation in general practice is limited, a knowledge gap that urgently needs further research. This study aims to examine the current approaches general practice uses for decarbonisation and to provide practical recommendations for facilitating, advancing, and sustaining these efforts, contributing to the goal of a net zero NHS.

**Methods and analysis:**

This 30-month longitudinal case study spans three geographical sites in England and uses a mixed methods study design. It adopts a comprehensive approach, merging sociological (Normalisation Process Theory - NPT) and behavioural theories (Theoretical Domains Framework - TDF) to understand and address factors influencing decarbonisation efforts in general practice. NPT focuses on collective behaviours, emphasising relationships and interactions among professionals, patients, and stakeholders. TDF, with 14 domains simplifying behavioural change theories, concentrates on individual, social, and environmental factors. A systematic review will be conducted, and quantitative and qualitative data will be collected over a 12-month period from general practice, staff, patients, public, and key stakeholders’ perspectives through surveys, interviews, and focus groups. Additionally, a non-clinical carbon calculator, alongside prescribing data, will be gathered to assess measurable changes in carbon emissions, informing a budget impact model for practice-specific and generic use.

**Dissemination:**

A dynamic dissemination and impact strategy is aimed at ensuring broad awareness, adoption, and accelerated uptake of decarbonisation measures. Outputs, including lay summaries, factsheets, policy briefs and academic presentations will be produced throughout the study and undergo regular review, targeting key audiences and ensuring alignment with regional and national goals.

## Plain Language Summary

The National Health Service (NHS) in the UK has set an ambitious goal to become a “net zero” healthcare system, meaning it aims to significantly reduce its carbon emissions. General practice, which includes GP surgeries and other local healthcare services, contributes a notable amount to these emissions. However, there is little information on how to successfully reduce these emissions in a sustainable way. This study seeks to explore how general practices are currently trying to cut their carbon footprint and will provide practical recommendations to help them do more and do it better.

The research will look at three areas in England and gather information over a year using a mix of surveys, interviews, and focus groups. These will involve healthcare staff, patients, the public, and other key individuals who are affected by or involved in these changes. Two different approaches will help guide the study, one focuses on how people work together and adapt to new ways of working, and the other looks at what motivates people to change their behaviour. The study will also measure the carbon emissions of practices using specific tools, such as a carbon calculator and data on medical prescribing, to see the impact of these efforts and estimate the costs.

The findings will be shared widely through various formats, including easy-to-read summaries, factsheets, and presentations. The goal is to ensure that everyone, from policymakers to the public, is aware of the work being done and can help make these changes happen faster and more effectively across the NHS.

## Introduction

The imperative to address climate change and reduce carbon emissions has become increasingly urgent, as underscored in the United Kingdom (UK) by the recent 'Delivering a 'Net Zero' NHS' report ( NHS, 2020). This report emphasises the critical need for all organisations within the National Health Service (NHS) to reduce their carbon emissions and incorporate sustainability into their practices. The NHS contributes 25% to all public sector carbon emissions, constituting 4–5% of the total UK carbon emissions ( Naylor & Appleby, 2012; NHS, 2020). Primary care, encompassing the direct delivery of care, staff and patient travel, commissioned health and care services, supply chain, infrastructure, and prescription practices, significantly contributes to this carbon footprint ( Tennison *et al.*, 2021).

While recognising the pivotal role of general practice ( BMA, 2021; Nunes *et al.*, 2016; Powell, 2021), the 'Net Zero' NHS report falls short in providing detailed guidance on how decarbonisation can be effectively achieved. This knowledge gap necessitates further research to elucidate the specific actions and strategies that can facilitate carbon reduction.

Growing recognition of general practice's role in addressing climate change is evident, with various resources emerging in the UK to support decarbonisation. These include the Green Impact for Health (GIFH) Toolkit ( SOS UK, 2024), non-clinical carbon calculator ( SEE Sustainability, 2024), decarbonisation guide ( SEE Sustainability, 2021), and the high-quality, low-carbon asthma toolkit ( Greener Practice, 2022) Professional bodies such as the Royal College of General Practitioners (RCGP) and the British Medical Association (BMA) also provide guidance on sustainability ( BMA, 2020; RCGP, 2022). Networks such as Greener Practice and initiatives like the RCGP Net Zero Hub further encourage action on sustainability in general practice ( Greener Practice, 2024). However, evidence is lacking on these resources’ utilisation, reach across practices, cost impact, and their effect on reducing carbon emissions.

The distributed organisational structure of general practice, with around 7,000 practices in 9,000 buildings in England alone, poses a significant challenge to achieving decarbonisation across the entire sector ( Wilkinson, 2021). Leadership, cultural, and behavioural changes are required at all levels within and outside healthcare systems ( Atwoli *et al.*, 2021). Achieving this demands coherent, collaborative, and locality-based yet nationally aligned action, planning, and delivery mechanisms ( Gudde *et al.*, 2021). Our study aims to contribute to addressing this by focusing on how decarbonisation can be effectively and practically implemented in general practice. In doing so, the study’s purpose is to provide evidence that can inform local, regional, national and international guidance, policy and practice.

### Why this research is needed now

The UK has taken an aspirational step by setting a goal to achieve a net zero health service, as outlined in the 'Delivering a 'Net Zero' NHS' report ( NHS, 2020). This commitment spans the three Greenhouse Gas Protocol (GHGP) scopes, encompassing direct emissions, indirect emissions from energy generation, and all other indirect emissions generated throughout the supply chain, including patient and visitor travel as well as medicines at home ( NHS, 2020).

The National Institute for Health Research Health Services and Delivery Research (NIHR HS&DR) call in 2022 ( NIHR, 2022) for research to support a net zero health and social care system reflected the critical importance of increasing the evidence base underpinning this area. Against the backdrop of the 2021/22 NHS Standard Contract ( NHS, 2021) requirement for Green Plans, our study is positioned to generate timely findings that can inform local, regional, national and international strategies. A notable gap is the absence of evidence on what is required to enable and support general practices to decarbonise ( O’Hare & Lyons, 2023). There have been several studies that have identified gaps in evidence relating to the efficacy of decarbonisation actions, best practice and patient perspectives ( Dupraz & Burnand, 2021; Kotcher *et al.*, 2021).

Despite the acknowledgment by professional bodies such as the RCGP and BMA, a survey commissioned by the Health Foundation found a lack of public recognition regarding the NHS's significant contribution to carbon emissions ( The Health Foundation, 2021). This emphasises the need for research to understand how patients’ views may facilitate or hinder actions toward decarbonisation. Public engagement and understanding patients' views constitute significant aspects of our study, ensuring a holistic exploration of the challenges and opportunities encountered in achieving net zero carbon emissions.

### The role of general practice in achieving a net zero NHS by 2040

The World Organization of Family Doctors (WONCA) has called for global commitment from general practitioners (GPs) to act on tackling climate change ( Wonca, 2019). General practice staff, positioned as agents of systemic and individual change ( Veidis *et al.*, 2019; Wonca, 2019), can play a pivotal role in addressing this issue through their connection with patients and communities ( Kemple, 2019; Xie *et al.*, 2018). Despite increasing calls for health professionals to address climate change, existing literature supporting such efforts is scarce, and interventions' effectiveness is underexplored ( Dupraz & Burnand, 2021).

A Lancet report ( Watts *et al.*, 2021) emphasises that mitigation efforts should actively generate health co-benefits, reduce environmental impacts, and maintain or improve the quality of care. However, knowledge on climate change mitigation and adaptation in primary care remains limited ( Kotcher *et al.*, 2021).

A comprehensive understanding of how various factors, including institutional, organisational, financial, professional, and patient aspects, either facilitate or inhibit the introduction and maintenance of these initiatives is needed. Our study is designed to address this by generating evidence and recommendations related to the multifaceted challenges and opportunities within general practice. It includes data collection from general practice staff, patients, and key stakeholders at local, regional, and national levels. This comprehensive approach aims to understand the necessary changes that are urgently needed in knowledge, practice and values across organisations to drive decarbonisation within the general practice system.

We are conducting a systematic review (i) to investigate the central research question of our study: ‘ *What institutional, organisational, professional, and patient factors affect the implementation and long-term sustainability of actions aimed at to reducing greenhouse gas emissions in general practice?’*. It is focusing on literature related to institutional, organisational, behavioural and systems change. PRISMA guidelines will be followed. The review has been registered with PROSPERO [CRD42023470889] and a systematic review protocol paper is currently under review for publication.

### Currently available resources to assist decarbonising general practices

Several tools and resources are currently available to assist general practices in making a substantial contribution to achieving decarbonisation. Notable among these is the RCGP’s GIFH toolkit developed in 2014 ( SOS UK, 2024), with approximately 1,100 general practices in the UK registered to use it. The toolkit provides 100 suggestions under key headings such as Water, Travel & Exercise, Quality Improvement, Zero Carbon, News and Communication, Food & Drink, Vulnerable Groups, Energy Saving, Healthy Planet, Waste & Recycling, Learning, and Social Prescribing.

The High-Quality and Low-Carbon asthma care toolkit ( Greener Practice, 2022), launched in 2022, aims to improve asthma outcomes while reducing carbon emissions. Despite having over 6,000 visits to its website since its launch, the changes made are unknown.

The Decarbonisation Guide ( SEE Sustainability, 2021) is available for a cost of £50, is accessible to all UK general practices via the SEE sustainability website.

However, the challenge lies in the lack of evaluation for these tools ( Powell, 2021). A recent BJGP editorial ( Kemple *et al.*, 2021) identified three crucial blocks to implementation and maintenance based on seven years' experience with the GIFH toolkit: general practices find it unsustainable or unreasonable to take on extra un-resourced work, a lone enthusiast in a practice may achieve little without wider support, and some practice teams make great progress with most tasks but struggle with complex challenges needed for more active decarbonisation. Addressing these challenges is likely to require new funding to support decarbonisation, wider support for individuals leading decarbonising actions, and collaborative efforts to learn from data, experience, and new ideas.

Our study focuses on bridging this gap by identifying how decarbonisation resources are being used, what motivates general practice teams to use them, and what is needed to enhance their overall impact on decarbonisation.

### Initial programme theory

**Figure 1.**
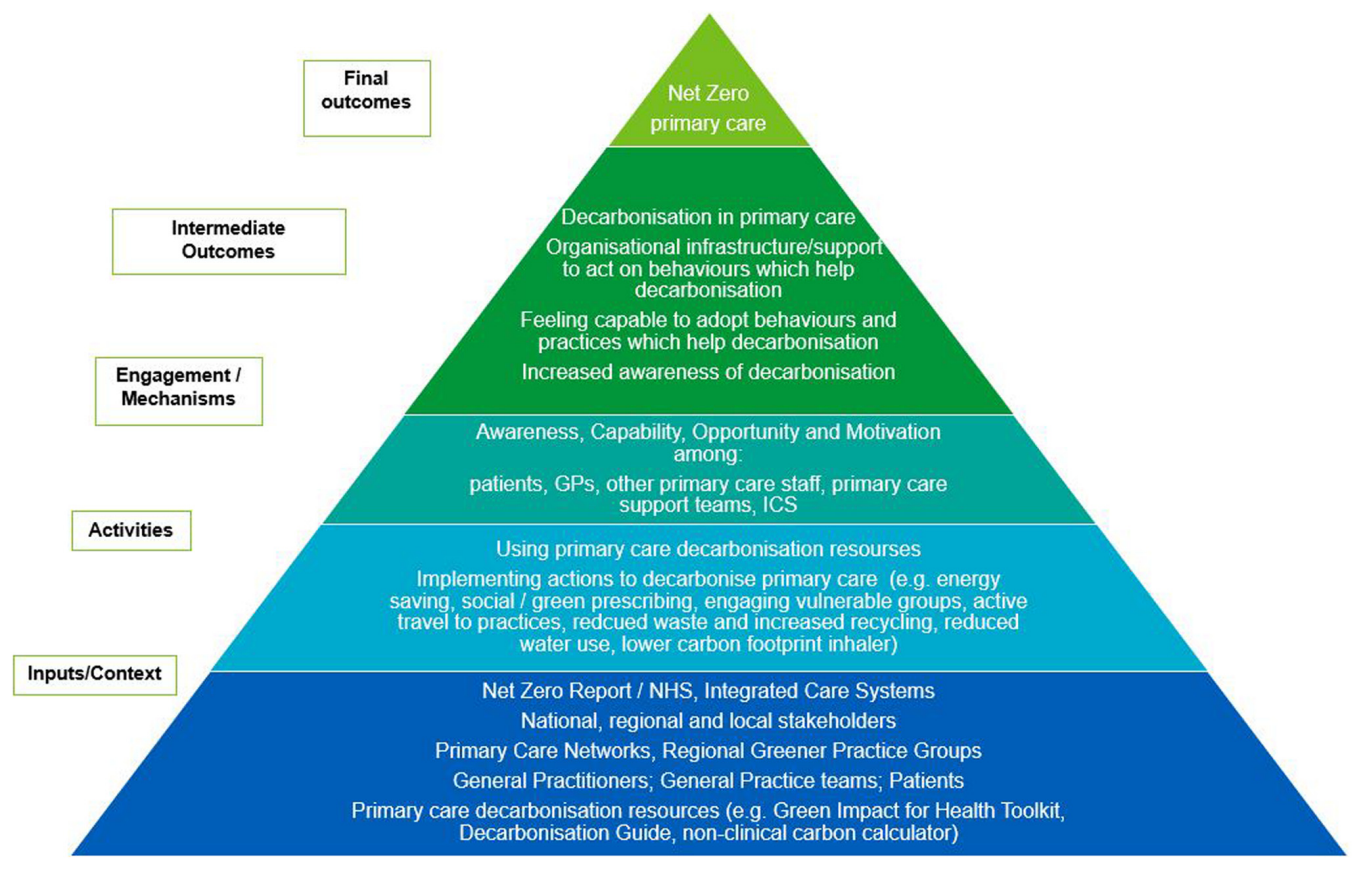
Initial Programme Theory (IPT) – hypothesised from primary care decarbonisation resources, literature and stakeholder and researcher insights.

In developing our study, we constructed an initial programme theory (IPT) to capture insights and evidence regarding efforts to decarbonise the health sector. This IPT outlines how interventions linked to currently available decarbonisation resources are introduced within organisational contexts, triggering mechanisms that lead to outcomes ( Figure 1).

Building on characteristics identified by Koleros and colleagues (2018), our IPT incorporates awareness, capability, opportunity, and motivation as key mechanisms. This theoretical framework serves as a foundation for understanding the complexities of decarbonisation efforts and informs the design of our study.

The findings of the systematic review will be used to refine the program theory, focusing on the mechanisms, including behaviours, processes, and activities, that contribute to the successful decarbonisation of general practices.

In summary, our study responds to the urgent need for evidence on how general practices can effectively contribute to achieving a net zero health service. By exploring the experiences and perspectives of general practice teams and patients, evaluating the use of existing resources, and informing national and local policies and strategies, our research aims to provide valuable insights into the challenges faced and opportunities available in decarbonising general practice.

## Aims and objectives

This study seeks to understand how general practice is implementing decarbonisation actions and develop practical recommendations to support, enhance, and expedite the implementation and sustainability of decarbonisation efforts in general practice, contributing to the achievement of a net zero NHS by exploring staff, patients’ as well as key stakeholders’ perspectives. More specifically its objectives include:

- the refinement of an initial programme theory, with identification of the elements of processes, behaviours, and activities that facilitate climate action in general practice; to explore institutional, organisational and professional factors that influence the implementation of decarbonisation actions;
- to explore the views of patients and public about the implementation of actions to decarbonise general practices, with particular attention to where such actions may affect the provision of care and/or health inequalities;
- to examine the cost implications involved in adopting different approaches to decarbonisation using a primary care budget impact model;
- to conduct key stakeholder (local, regional and national policy, commissioning and general practice) exploration of views about how to support actions to decarbonise general practices; and
- to synthesise and integrate all findings into a programme theory that draws together the factors and mechanisms that influence the implementation and sustainability of actions to decarbonise general practices and develop the respective recommendations.

## Methods

HRA and Health and Care Research Wales (HCRW) Approval has been given for the study (REC reference: 23/PR/1169) on 6 ^th^ November 2023.

### Patient and public involvement statement

Our study was developed with extensive patient and public involvement and has two lay representative co-investigators and a patient and public panel made up of nine individuals who are providing advice and feedback on all aspects of the study.

The study comprises a mixed methods 12-month longitudinal study in which the implementation of decarbonisation initiatives will be evaluated by drawing on the NPT and TDF frameworks, alongside a systematic review (PROSPERO [CRD42023470889]) and collecting economic data that will be used to inform the development of a budget impact model. This integrated approach aims to provide a robust framework for understanding, analysing and promoting the successful implementation and sustainability of decarbonisation activities in general practice. A mixed methods study design is used which adopts a comprehensive approach, integrating sociological theories (Normalisation Process Theory – NPT ( Finch *et al.*, 2015; Murray *et al.*, 2010)) and behavioural theories (Theoretical Domains Framework – TDF ( Atkins *et al.*, 2017) to understand and address the complex factors influencing these efforts. This combination allows for a systematic exploration of individual and group determinants, facilitating the identification of cognitive, affective, and environmental factors crucial for planning and implementing decarbonisation activities in general practices.

NPT will be valuable in understanding collective behaviours, emphasising the importance of relationships and interactions among general practice professionals, patients and key stakeholders ( Finch *et al.*, 2015; Murray *et al.*, 2010). It suggests that successful interventions are more likely when practice staff value the intervention, commit to engagement, allocate resources collectively, and find the actions useful. The TDF, with its 14 domains simplifying various behavioural change theories, focuses on individual, social, and environmental factors relevant to engaging general practice teams in decarbonising general practices ( Atkins *et al.*, 2017).

### Setting

The study focuses on three Integrated Care Board (ICB) areas: Coventry and Warwickshire, Birmingham and Solihull, and South Yorkshire. These regions have been chosen due to their varying levels of engagement with decarbonisation initiatives. Each area has expressed support for the study and demonstrated enthusiasm to participate through letters of support.

Coventry and Warwickshire ICB includes 119 general practices serving a population of 1.02 million ( NHS Coventry & Warwickshire, 2023). Birmingham and Solihull ICB encompasses 184 general practices, catering to a population of 1.3 million ( NHS Birmingham & Solihull, 2023). South Yorkshire ICB comprises 170 general practices, serving a population of 1.4 million ( NHS South Yorkshire (2024). In total, there are 473 general practices across these three areas.

The population sizes and characteristics of these areas vary, reflecting diverse healthcare needs and demographic profiles. The inclusion of these areas in the study ensures a comprehensive understanding of the challenges and opportunities associated with decarbonisation across different contexts.

### Recruitment

***General practices and staff.*** All general practices in each of three study site areas will be invited to take part in an online survey to ascertain their current engagement in decarbonisation efforts. Subsequently, 12 practices will be purposively sampled from the survey responses, with a goal of selecting four from each study site. The recruitment strategy aims for diversity across characteristics such as list size, number of GPs, level of deprivation, percentage of patients from ethnic minority groups, location (rural, semi-rural, urban), and engagement with decarbonisation activities (low, medium, high).

**Figure 2.**
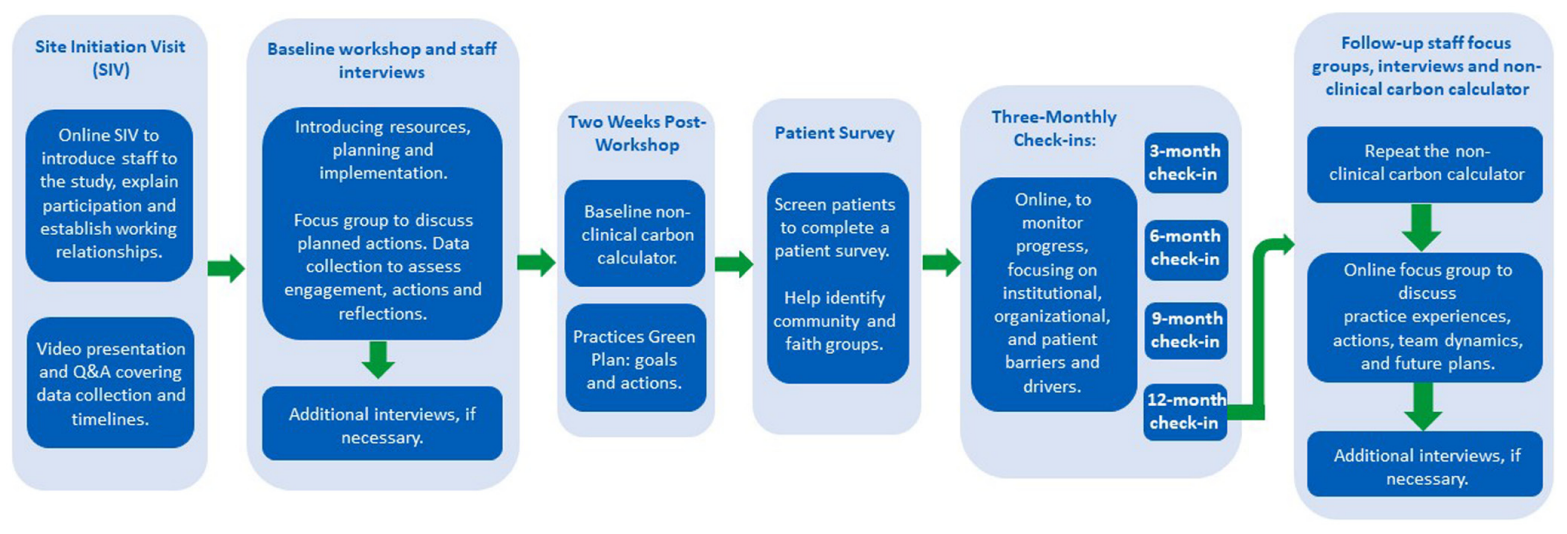
Flowchart representation of the activities that each practice will aid and/or undertake throughout the longitudinal study.

Staff from the selected case study general practices will be invited to participate in a baseline workshop (which will include a presentation from the research team followed by a facilitated team discussion with additional and staff interviews if required), two weeks check-in for confirming the general practice has drafted its green action plan, three-monthly check-ins and follow-up focus groups and staff interviews (see Figure 2). These interactions will explore practice staff members’ awareness of decarbonisation initiatives, the challenges and opportunities they perceive.

Figure 2 below, illustrates the activities undertaken by each practice, providing a concise summary of practice elements throughout the entire longitudinal study. The key elements include various stages, interventions, and milestones that contribute to the comprehensive understanding of the practice's engagement and progression over time.

***Patients and public.*** Within each case study practice, a database search will be undertaken to identify a representative sample of adult patients who have had recent contact with the practice. These patients will be provided with a participant information leaflet, inviting them to actively engage in the study by completing a survey, and taking part in a follow up interview. To ensure a diverse representation, community and faith groups in the vicinity of the practice will also be identified and members will be invited to complete a survey, and taking part in a follow up interview, particularly those targeting under-served populations (see Figure 2). This inclusive approach aims to broaden the perspectives gathered within the study.

***Key stakeholders.*** This will involve local (i.e. in the vicinity of each of the case study practices), regional and national key stakeholders, with recruitment being purposive to ensure a diverse range of participants, to understand and improve the implementation and sustainability of decarbonisation actions in general practice. This will include professional opinion leaders, policy makers, commissioners, educators and patient representatives.

### Sample sizes

***General practices and staff.*** All general practices within the three study sites, totalling 473 practices, will receive an invitation to participate the survey. We aim for a minimum response rate of 30%, equating to 142 practices, from which we will recruit 12 practices to become case studies (see below).

Sample sizes for staff interviews and focus groups are determined with the aim of achieving maximum variation. Members of staff in the selected case study general practices will be invited to take part in the focus group and interviews; the sample will be up to 5 members of staff in each general practice (n=60).

***Patients and public.*** We will aim for survey responses from 35 patients at each practice, totalling at least 400 across the 12 case study sites. Anticipating a response rate of approximately 20%, we will initially invite 150 patients at each practice and extend further invitations if needed. To ensure a diverse participant sample, we will specifically target the recruitment of under-served populations residing in the vicinity of the participating practices, with the aim of recruiting an additional 100 participants.

We plan to purposively select 10–12 patients per study site to participate in an interview, totalling 30–35 participants. This selection aims to ensure diversity across age, gender, ethnicity, socio-economic status, health status and general practice, specifically targeting survey respondents. To enhance representation, additional targeted invitations for interviews will be extended through general practice if certain demographic groups are not adequately represented among survey respondents.

***Key stakeholders.*** We will interview up to 15 regional and national policy stakeholders who have wider insight and 20 local stakeholders who can comment on ‘grassroots’ policy. We will also explore their views about how to support the implementation and sustainability of actions to decarbonise general practice.

### Data collection

***General practices and staff.*** The general practice survey will assess whether and how decarbonisation actions are currently occurring in general practices. Additionally, the survey explores awareness of available decarbonisation resources within general practice and identifies intentions, actions, and plans in place.

The survey will be accessible through an online platform (i.e. Qualtrics), featuring multiple-choice tick-box answers for efficiency and free-text questions for additional comments, and is designed to take 5-minutes to complete. The survey will also assess expressions of interest in participating in the 12-month longitudinal study where practices will be asked to implement a decarbonisation plan over 12 months (see below).

All interviews and focus groups will be conducted either by telephone or online, offering flexibility to participants while avoiding the carbon from travel. The data generated from these interactions will be digitally recorded and transcribed verbatim to ensure an accurate record of participants' perspectives.

A non-clinical carbon calculator ( SEE Sustainability, 2024) and publicly available prescribing data to estimate emissions associated with the practice's prescribing of inhalers will be used at baseline (completed two weeks post-workshop) and after 12 months to assess measurable changes (see Figure 2). The data collection process will provide useful insights, considering the current pressures on general practices.

A health economics analysis will assess the financial implications of decarbonisation strategies in general practice by creating a detailed budget impact model, developed in Microsoft Excel. This model will establish baseline costs and resource utilisation under various categories contributing to carbon emissions, derived from existing resources and survey responses. Practice specific models for the 12 participating practices will be developed, incorporating baseline data and applying scenarios of potential interventions, providing practices with information on changes and associated cost implications over a 12-month period. Data on changes made by each practice over 12-months will be inputted into the model. A user-friendly generic budget impact model will also be developed for widespread use in general practice and to inform further stages of the study, allowing for extensive sensitivity analyses across various scenarios and assumptions. The data collection process from practices will be comprehensive but will be flexible enough to accommodate current pressures on general practices.

***Patients and public.*** The survey will have a maximum of 20 questions to collect participants' demographics, views on climate change, awareness of their practice's decarbonisation initiatives and concerns about the impact on patient care. Participants will rank their agreement with statements and have the option to provide additional comments. The survey will ascertain interest in participating in an interview. Patients and community groups survey data will be collected online or on paper and stored securely.

The patient interview topic guide will elicit patients' views about general practice sustainability, practice-initiated actions and concerns about the impact on patient care. The interview will be offered both via the telephone or online videocall as per their preference. Data will be recorded digitally and transcribed verbatim. Interviews will last up to 30 minutes and will be audio-recorded. The NICE report, ‘Public dialogue on environmental sustainability’ shall be considered when developing patient and public survey and interview topic guides ( NICE, 2023).

***Key stakeholders.*** Interviews will be conducted through video meetings or telephone conversations. These interviews aim to capture insights on how local, regional, and national strategies, policies and plans are directed toward decarbonising general practice and achieving NHS net zero goals. Participants will also be asked to share their experiences regarding the facilitators and barriers influencing the reach and adoption of these initiatives.

### Data analysis

***Quantitative data.*** General practices and staff survey data will be examined using descriptive statistics to describe the extent to which practices are integrating or contemplating decarbonisation efforts. Associations between practice characteristics (e.g., rural/urban designation, size) and the levels of interest in and experience with decarbonisation activities will be explored using the statistical software package SPSS ( IBM, 2020), employing descriptive statistics. These data will be instrumental in characterising the sample, describing age, socio-demographic details, ethnicity, and other relevant factors for both staff and patients. Chi-square tests will be employed to investigate associations between patient and staff characteristics and their perspectives on general practices’ decarbonisation. Consideration will be given to multivariate modelling.

The patient and public survey data will undergo analysis using SPSS ( IBM, 2020). This analysis will involve descriptive statistics, conducted both at the practice/site level and across the entire sample. To examine the associations between patient characteristics (such as gender, age, ethnicity, and registered practice) and their perspectives on general practice decarbonisation, chi-square tests will be used. If appropriate to the dataset, multivariate modelling will be undertaken to rigorously test and explore the interplay between variables.

***Qualitative data.*** Interview data will be transcribed verbatim to ensure a thorough record of participants' responses. The qualitative data obtained from these transcripts will undergo analysis using a Framework approach ( Gale *et al.*, 2013). The coding framework will be constructed by drawing on both the NPT and TDF coding dictionaries, incorporating inductive and deductive elements guided by the constructs of NPT and TDF ( Atkins *et al.*, 2017; Finch *et al.*, 2015; Murray *et al.*, 2010). This will enable us to explore the data from the specific perspectives provided by NPT and TDF and subsequently integrate the findings cohesively. To streamline the coding process, a coding manual will be developed. This will ensure a thorough examination and interpretation of interview data, facilitating a richer understanding of the factors influencing perceptions and experiences related to decarbonisation efforts within general practice.

### Data synthesis

We will synthesise and integrate all findings into a revised programme theory that draws together the factors and mechanisms that influence the implementation and sustainability of actions to decarbonise general practices and develop implementation and sustainability recommendations.

This will involve cumulatively integrating findings using triangulation, cross-verifying data sources and using mixed methods matrices to map and compare results ( O’Cathain *et al.*, 2010). Drawing on the above we will assess contextual factors and mechanisms affecting the adoption of decarbonisation actions by organisations and workforce. The synthesis will refine the initial program theory ( Figure 1), informing preliminary recommendations and potential interventions for decarbonisation adoption and maintenance. Research gaps will be identified and detailed throughout the study. They will be highlighted in dissemination plans, addressed in stakeholder consensus workshops, engaging key representatives to discuss findings, implications, and prioritise recommendations for national policy and practice transformations, facilitated by the Nominal Group Technique ( Delbecq *et al.*, 1975).

## Ethics and consent

HRA and Health and Care Research Wales (HCRW) Approval has been given for the study (REC reference: 23/PR/1169) on 6 ^th^ November 2023. Ethical review was undertaken by the Research Ethics Committee (REC) and an Assessment to check compliance with the UK Study-wide governance criteria, as well as relevant additional nation specific areas of review.

Written and verbal consent will be obtained from all participants involved in the study. Written consent will be sought for participants where feasible, ensuring that they fully understand the purpose of the study, their role, and their rights. Where written consent is not practicable such as during remote participation, verbal consent will be obtained. The use of verbal consent was approved by the ethics committee.

## Strengths and limitations of this study

### Strengths

- Longitudinal case study across multiple sites and general practices.
- Pragmatic study design and data collection.
- Using mixed methods and theoretical frameworks.
- Inclusion of stakeholders from devolved nations.

### Limitations

- Collecting data from busy general practices can be challenging.
- Study settings are within England, presenting a limitation when applying and generalising findings to other settings.
- Participant attrition.

## Dissemination and impact

We have set up a stakeholder advisory group, and we have two lay representative co-investigators and patient and public panel to ensure that we generate and disseminate findings in a way that is likely to maximise impact.

At the start of the study, we will initiate a dynamic and comprehensive dissemination and impact strategy with a primary goal of fostering awareness, adoption, and swift integration of measures aimed at reducing the environmental impact linked to general practice. This strategy encompasses a wide array of deliverables, ranging from lay summaries, knowledge mobilisation factsheets, and policy briefs to materials on the study’s website, social media content, press releases, as well as academic presentations and publications. The research management team will conduct a thorough review of the strategy every three months to ensure that the outputs align with key findings, with a particular emphasis on clearly communicating these findings and recommendations to regional and national audiences.

The primary target audiences for this study include commissioners and NHS managers, general practices, and primary care networks. Additionally, we aim to engage patients and the public, external statutory organisations, non-statutory bodies, and the academic community, particularly those focused on primary care. Following the publication of the study protocol paper, we anticipate the submission of a minimum of five open-access papers to high-impact peer-reviewed journals. This process will culminate in the publication of the program theory, accompanied by evidence-based recommendations designed to enhance the adoption and utilisation of decarbonisation resources within general practice. The focus of all outputs will be on providing evidence about what is required to implement, embed, and integrate (or normalise) actions in general practice to achieve decarbonisation.

## Data Availability

No data are associated with this article.

## Software availability statement

For the statistical analyses conducted in this study, SPSS was used. As an alternative, a free open-source software option capable of performing equivalent functions include PSPP.
