# Supplementary material for: Supporting the drive for net zero by decarbonising general practice – A longitudinal study protocol
Source: NIHR Open Res. 2025 Sep 24;4:82. Originally published 2024 Dec 24. [Version 2] doi: 10.3310/nihropenres.13833.2 (PMC12800612; doi:10.3310/nihropenres.13833.2)
Supplement: Peer review history

# Reviewer report: Reviewer response for version 2

**DOI:** 10.3310/nihropenres.15357.r39032

**Published:** 2026-01-13

**Authors:** Emilio Sessa

**Affiliations:** 1 University of Palermo, Viale delle Scienze, Italy

This article presents a study protocol for a mixed-methods, longitudinal, multi-site investigation examining how decarbonisation actions can be implemented and sustained within UK general practice. The study is positioned within the broader policy imperative of achieving a net zero NHS by 2040, responding directly to NHS England strategy, the NIHR HS&DR research agenda, and growing professional calls for climate action in healthcare.

 The authors propose a theory-informed design, integrating:

- A registered systematic review (PRISMA-aligned, PROSPERO registered),
- A large cross-sectional survey of general practices,
- Longitudinal case studies in 12 purposively sampled practices across three ICBs,
- Patient and public surveys and interviews (with attention to underserved groups),
- Key stakeholder interviews (local to national),
- A health-economic budget impact model, and
- Synthesis through refinement of an Initial Programme Theory (IPT), drawing on Normalisation Process Theory (NPT) and the Theoretical Domains Framework (TDF).

The study aims to generate actionable evidence on institutional, organisational, professional, and patient-level factors influencing decarbonisation in primary care, culminating in policy-relevant recommendations.

 Overall, the protocol is ambitious, timely, and highly relevant to current NHS priorities.

 The study is fundamentally sound in intent and design, but there are important conceptual and methodological ambiguities that must be addressed to ensure scientific robustness, interpretability, and downstream usability of findings.

 The protocol repeatedly refers to “decarbonisation actions” without clearly specifying:

- What counts as a *decarbonisation action*,
- Whether actions are expected to lead to measurable GHG reductions, or
- Whether symbolic, behavioural, or preparatory actions are treated as equivalent to structural emissions reductions.

This creates a serious construct validity problem.

 For example:

- Installing LED lighting,
- Switching inhaler types,
- Running staff awareness sessions,
- Producing a Green Action Plan,

are treated as conceptually equivalent, despite radically different emissions impacts.

 As far as the conceptual ambiguity around decarbonization is concerned, the authors should:

1. Explicitly define “decarbonisation actions”, distinguishing at minimum between:
  - Behavioural/process actions (e.g. education, prescribing choices),
  - Operational actions (energy, travel, procurement),
  - Structural/systemic actions (estate changes, commissioning, contracts).
2. Clarify whether the study evaluates:
  - Implementation *regardless of carbon impact*, or
  - Implementation *weighted by emissions relevance*.
3. Introduce a simple typology or hierarchy (even qualitative) linking actions to:
  - Scope 1, 2, and 3 emissions,
  - Estimated order-of-magnitude impact (low / medium / high).

This can be done without full carbon accounting, but must be conceptually explicit.

 As far as the economic component is concerned, the authors should:

1. Explicitly state the decision problem the model addresses (e.g. “What does it cost a typical practice to implement common decarbonisation actions?”).
2. Clarify whether the model will:
  - Compare scenarios,
  - Identify cost drivers,
  - Support commissioners, or
  - Support practices.
3. Explicitly acknowledge what the model does NOT do, e.g.:
  - It does not estimate cost-effectiveness per tCO₂e avoided,
  - It does not replace full LCA.
4. Add a short paragraph justifying why a budget impact model (rather than cost-effectiveness analysis) is the appropriate tool at this stage.

The study protocol is methodologically credible, policy-relevant, and ethically sound, but requires conceptual tightening and clearer analytical framing to ensure that its findings will be interpretable, defensible, and genuinely useful for advancing decarbonisation in general practice.

Is the study design appropriate for the research question?

Yes

Is the rationale for, and objectives of, the study clearly described?

Yes

Are sufficient details of the methods provided to allow replication by others?

Partly

Are the datasets clearly presented in a useable and accessible format?

Partly

Reviewer Expertise:

Climate neutrality

I confirm that I have read this submission and believe that I have an appropriate level of expertise to confirm that it is of an acceptable scientific standard, however I have significant reservations, as outlined above.

# Reviewer report: Reviewer response for version 1

**DOI:** 10.3310/nihropenres.15031.r36073

**Published:** 2025-08-14

**Authors:** Beata Gavurova

**Affiliations:** 1 Technical University of Košice, Košice, Košice Region, Slovakia; 2 Tomas Bata University in Zlin, Zlin, Czech Republic

The manuscript deals with supporting the way to net zero in the field of decarbonising. It is presented in a form of the study protocol. The Introduction section represents a standard entrance into the discussed topic for ordinary reader. The last subsection involved is called Initial programme theory. As it is written in the manuscript, it is constructed by the authors. Hence, it is an outcome of their work, but unexpectedly, it is included in the Introduction section. This has to be shifted into the section, where the suggestions from the analytical section are presented. On the other hand, in a form as it is written now, it is not based on any outcomes from the analytical processing.

 The Aims and objectives section is quite rich and hence, it lists the four aims, which are quite particular, but they are nowhere mentioned to be fulfilled or not fulfilled in the remaining sections of the manuscript. It is needed to evaluate these aims in the manuscript.

 The Methods section introduces the setting of the research performed. There is no information on representativeness of the respondent selection. The last two paragraphs of the Recruitment section look like extracted from elsewhere.

 The following subsection Sample sizes describes the amounts of practices, patients, and case study sites. Why did you select just right such numbers? It is nowhere explained, why you selected just right 473 practices, 35 patients, and 12 case study sites. If you presume a response rate at a level of 20 %, resulting in 30 to 35 patients at each site, then 150 patients at each practice would not mean 400 ones across all the sites. This number would be much higher. The original invited number should be higher than 150 at each practice. It is also not clear whether additional recruitment of 100 participants is meant at each site or altogether.

 There is mentioned the budget impact model developed in Microsoft Excel in the Data collection section. Is it already invented and prepared for use? Is it your invention. Such information is very missing in the text of the manuscript. What does it mean that data at each practice will be inputted through a 12-month period? If you plan to ask several tens of patients through a 5-minute survey, then a period of 12 monts is very lengthy and redundant.

 In the Data analysis section, you mention you will use the SPSS software package in order to explored decarbonisation activities. It is unclear, why the budget model invented in Microsoft Excel is not listed here. Will you not use it? It makes no sense related to the above-mentioned facts related to the budget model. Successively, you mention qualitative data that will be observed. It is a pity you will not use the software package also here. Such an approach is little but strange as you have different approaches to various observed data, but they are not explained. Unification of all the techniques applied will make the outcomes more versatile.

 The last section expresses that you plan to publish at least studies based on the survey. Nevertheless, there is no mention about their contents or mutual differentiation. The five studies based on the same basement with the same techniques are literally unacceptable.

 The biggest shortcoming of the whole study is a missing questionnaire. The whole study is written as you are going to launch your survey just right now, but you have not revealed questionnaire and even the techniques that will be applied. These are the crucial points. Therefore, it is required to revise the manuscript in order to offer reader this information and to point out to all the above-listed points, otherwise, such a study protocol is useless and redundant.

Is the study design appropriate for the research question?

Partly

Is the rationale for, and objectives of, the study clearly described?

Partly

Are sufficient details of the methods provided to allow replication by others?

No

Are the datasets clearly presented in a useable and accessible format?

No

Reviewer Expertise:

economics, digitalisation, circular economy, SDGs, regional disparities

I confirm that I have read this submission and believe that I have an appropriate level of expertise to confirm that it is of an acceptable scientific standard, however I have significant reservations, as outlined above.

# Response

**Published:** 2025-09-19

**Authors:** Ana Raquel Nunes

**Affiliations:** University of Warwick Medical School, UK

The authors thank the reviewer for all their comments and address specific questions below.

 - The initial programme theory represents a conceptual starting point of our work and as such is presented as a starting framework to be refined during the study’s implementation through subsequent data collection and analysis.

 - As this is a study protocol, the aims cannot be yet evaluated for fulfilment.

 - Our sampling strategy is purposive and designed to maximise diversity in practice characteristics, patient populations, and geographical areas, rather than statistical representativeness. The following sentence has been added: “The recruitment strategy was designed to ensure diversity across key characteristics. Sample sizes were determined to maximise variability and capture a broad range of perspectives rather than achieve statistical representativeness.”

 - We have clarified and expanded this section. The following has been revised and added: “ ***General practices and staff.*** All general practices within the three ICB areas, totalling 473 practices, will receive an invitation to participate the survey. We aim for a minimum response rate of 30%, equating to 142 general practices, from which we will recruit 12 general practices to become case studies (see below).

 Sample sizes for staff interviews and focus groups will be determined with the aim of achieving maximum variation. Members of staff in the 12 general practices will be invited to take part in the focus group and interviews; the sample will be up to 5 members of staff in each general practice (n=60).

***Patients and public.*** We will aim for survey responses from 35 patients at each general practice, totalling at least 420 across the 12 general practices. Anticipating a response rate of approximately 20%, we will initially invite 175 patients at each general practice and extend further invitations if needed. Additionally, to ensure diversity within the sample, we will target the recruitment of under-served populations residing in the vicinity of the 12 general practices through 12 community and faith groups, with the aim of recruiting an additional 120 participants (i.e. 10 per community and faith groups).

 We plan to purposively interview 2–3 patients per general practice and/or community and faith groups to participate in an interview, totalling 24–36 participants. This selection aims to ensure diversity across age, gender, ethnicity, socio-economic status, health status and general practice, specifically targeting survey respondents. To enhance representation, additional targeted invitations for interviews will be extended if certain demographic groups are not adequately represented among survey respondents.

***Key stakeholders.*** We will interview up to 15 regional and national policy stakeholders who have wider insight and 20 local stakeholders who can comment on ‘grassroots’ policy. We will also explore their views about how to support the implementation and sustainability of actions to decarbonise general practice.

 The sample sizes strategy was designed to ensure diversity across key characteristics. Sample sizes were determined to maximise variability and capture a broad range of perspectives rather than achieve statistical representativeness.”

 - We will expand the description. The following sentences have been revised and added: “A health economics analysis will assess the financial implications of decarbonisation strategies in general practice by creating a detailed budget impact model, developed in Microsoft Excel. The budget impact model will be developed by the research team, informed by existing economic modelling approaches. Data will be collected at baseline (completed two weeks post-workshop) and after 12 months to assess measurable changes (see Figure 2). This model will establish baseline costs and resource utilisation. Practice specific models for each of the 12 participating general practices will be developed. A user-friendly generic budget impact model will also be developed for widespread use in general practice and to inform further stages of the study, allowing for extensive sensitivity analyses across various scenarios and assumptions. It will be piloted before full implementation. The data collection process from practices will be comprehensive but will be flexible enough to accommodate current pressures on general practices. The data will be collected prospectively over a 12-month period to capture seasonal variation in practice activity and energy use, which are important for understanding decarbonisation impacts. The data collection process will provide useful insights, considering the current pressures on general practices.”

 - The text has been revised to explain that SPPS will be used for quantitative survey data analysis, while the Excel-based budget impact model constitutes a separate tool specifically for economic evaluation. For the qualitative data, we will state that analysis will be undertaken using framework analysis, supported by qualitative analysis software (i.e., NVivo).  

 - A dissemination section has been added to make clear that each publication will present distinct analyses addressing different aspects of the study (e.g., survey findings, budget modelling, case studies, patient interviews, stakeholder interviews).

 The following has been added: “ **Dissemination**

 Findings and tailored recommendations will be shared directly with participating general practices to support their ongoing decarbonisation efforts. Dissemination will target a broad audience through academic journals, conferences, policy briefs, and knowledge mobilisation factsheets. Particular attention will be given to reaching stakeholders beyond primary care and the healthcare sector, recognising the wider applicability of the findings to other domains facing similar challenges.”

 - As this is a study protocol, the finalised questionnaire/survey cannot yet be included. This will be included in the article presenting the questionnaire/survey findings.

 - The study protocol paper has been revised accordingly.

# Reviewer report: Reviewer response for version 1

**DOI:** 10.3310/nihropenres.15031.r34456

**Published:** 2025-02-03

**Authors:** Loïc Lannelongue

**Affiliations:** 1 University of Cambridge, Cambridge, UK

This article presents the study protocol to examine the current approaches to decarbonisation of general practices and provide recommendations to reach the net zero target of the NHS. 

 This is a much needed and timely work, as there is a need for more evidence on how individuals and organisations engage with net zero strategies, and how such strategies can be improved to be more successful. 

 The rationale is clear, and the protocol well detailed, with a particular focus on patients engagement.

 Minor suggestions:

 - The justification for the different choices of sample sizes are not explained and could be added. 

 - Is there a plan to feed back recommendations on decarbonisation to participating GP practices?

 - Dissemination, I'd imagine that such work (methods and results) would also be of great interest outside of the primary care sector (or outside of the healthcare sector even), as the lot of challenges in terms of engagement would be similar. It would be worth considering a broader dissemination for that reason.

Is the study design appropriate for the research question?

Yes

Is the rationale for, and objectives of, the study clearly described?

Yes

Are sufficient details of the methods provided to allow replication by others?

Yes

Are the datasets clearly presented in a useable and accessible format?

Not applicable

Reviewer Expertise:

Sustainable research, green computing, health data science

I confirm that I have read this submission and believe that I have an appropriate level of expertise to confirm that it is of an acceptable scientific standard.

# Response

**Published:** 2025-09-19

**Authors:** Ana Raquel Nunes

**Affiliations:** University of Warwick Medical School, UK

The authors thank the reviewer for all their comments and address specific questions below.

 - The following sentence has been added: “The sample sizes strategy was designed to ensure diversity across key characteristics. Sample sizes were determined to maximise variability and capture a broad range of perspectives rather than achieve statistical representativeness.”

 - Yes. The following sentence has been added: “Findings and tailored recommendations will be shared directly with participating GP practices to support their ongoing decarbonisation efforts.”

 - The following sentence has been added: “Dissemination will target a broad audience through academic journals, conferences, policy briefs, and knowledge mobilisation factsheets. Particular attention will be given to reaching stakeholders beyond primary care and the healthcare sector, recognising the wider applicability of the findings to other domains facing similar challenges.”
